# Maternal characteristics associated with ovalbumin concentrations in breast milk

**DOI:** 10.1111/pai.70365

**Published:** 2026-05-13

**Authors:** Sophie A. Hughes, Jessica R. Metcalfe, Sharon L. Perrella, Donna T. Geddes, Debra J. Palmer

**Affiliations:** ^1^ The Kids Research Institute Australia The University of Western Australia Nedlands Western Australia Australia; ^2^ School of Molecular Sciences The University of Western Australia Crawley Western Australia Australia; ^3^ ABREAST Network Perth Western Australia Australia; ^4^ Centre for Human Lactation Research and Translation The University of Western Australia Crawley Western Australia Australia; ^5^ Department of Immunology Perth Children's Hospital Nedlands Western Australia Australia; ^6^ School of Medicine The University of Western Australia Crawley Western Australia Australia

**Keywords:** breast milk, egg, fish oil, gestational diabetes mellitus, human milk, lactation, maternal characteristics, ovalbumin

## Abstract

**Background:**

Breast milk ovalbumin concentrations have been measured with considerable inter‐individual variation. Body mass index (BMI) and gestational diabetes mellitus (GDM) in humans, and fish oil supplementation in pigs, have been associated with breast milk protein concentrations. This study aimed to identify whether breast milk ovalbumin concentrations are associated with these maternal characteristics.

**Methods:**

This is a secondary analysis of data collected during a randomized trial. Breast milk ovalbumin concentrations were measured in 66 atopic women 2–6 h after consumption of one egg at 2, 4, and 6 weeks of lactation. Associations between ovalbumin concentrations and maternal characteristics (BMI, GDM and fish oil supplementation during pregnancy) were investigated.

**Results:**

Participants with GDM (*n* = 6) had higher ovalbumin concentrations at 2 weeks of lactation (median = 0.87 [IQR 0.27–2.17] ng/mL) compared to participants without GDM (*n* = 60, median = 0.15 [IQR 0.05–0.67] ng/mL; *p* = .03). Participants who consumed fish oil supplements during pregnancy (*n* = 16) had higher ovalbumin concentrations at 4 weeks (median = 0.39 [IQR 0.07–1.20] ng/mL) and 6 weeks (median = 0.29 [IQR 0.15–0.86] ng/mL) compared to those who did not (*n* = 50, median = 0.05 [IQR 0.05–0.44] ng/mL at 4 weeks, *p* = .01; and 0.05 [IQR 0.05–0.40] ng/mL at 6 weeks, *p* = .01). Pre‐pregnancy BMI was not associated with ovalbumin concentrations.

**Conclusions:**

GDM and pregnancy fish oil supplementation were associated with higher breast milk ovalbumin concentrations. Future, larger studies should further investigate associations between these maternal characteristics and breast milk food protein concentrations.

**Key message**

Higher breast milk ovalbumin concentrations were found in women who had gestational diabetes mellitus and in women who took a fish oil supplement during pregnancy. These novel findings begin to explain previously observed inter‐individual variations in food allergen concentrations in breast milk. The influence of maternal characteristics on breast milk food allergen concentrations is important for the future translation of current maternal diet during lactation and child food allergy prevention trials.

## 1 INTRODUCTION

Common food allergens, such as cow's milk, egg, peanut and wheat, have been detected in breast milk at highly variable inter‐individual concentrations.^1^, ^2^ These inconsistent breast milk concentrations have been measured even after lactating women have fasted prior to and then ingested the same amount of food allergen.^3^, ^4^, ^5^, ^6^ In a study by Host et al,^3^ all participants consumed 500 mL of cow's milk; however, breast milk beta‐lactoglobulin concentrations ranged from 0.9 to 150 ng/mL. In another study by Palmer et al.,^4^ all participants consumed an identical muffin containing one cooked egg, and the breast milk ovalbumin concentrations varied from 0.1 to 29.8 ng/mL. These variations in breast milk concentrations may be due to maternal characteristic differences amongst lactating women. A recent systematic review^2^ concluded that new studies should investigate whether food allergen breast milk concentrations are associated with any specific maternal characteristics. Understanding the potential influence of these maternal characteristics will be valuable in refining future interpretation of results from current large‐scale, randomized controlled trials investigating whether the risk of developing food allergies in children can be reduced by maternal diets rich in food allergen consumption during lactation.^7^, ^8^

Proteins found in breast milk are primarily manufactured within the mammary glands; however, some breast milk proteins, such as immunoglobulins and food proteins, enter breast milk via the maternal circulation. In our search to identify influences on breast milk food protein concentrations, we started with consideration of maternal characteristics that may influence breast milk total protein composition. Stage of lactation influences breast milk protein content, with colostrum (1–3 days postpartum) having a higher protein content of 2.0 g/100 mL, compared to 1.8 g/100 mL in transitional milk (3 days to 2 weeks), and 1.2 g/100 mL by 10–12 weeks in mature milk.^9^ Several studies have identified that breast milk protein concentrations are higher in women with higher body mass index (BMI).^10^, ^11^, ^12^ Higher breast milk protein concentrations were measured in 70 non‐smoking women (with an average stage of lactation of 1–3 months) who had a BMI ≥28 kg/m^2^ especially when compared to women who were underweight with a BMI <18.5 kg/m^2^ (*p* = .006).^12^ In addition, higher concentrations of specific whey proteins (alpha‐lactalbumin and lactoferrin) and casein proteins (α_S1_‐casein and β‐casein) were also found in women with higher BMI.^12^ In addition, a systematic review and meta‐analysis (9 studies, *n* = 1057) has identified that women with GDM had higher breast milk protein concentrations in both colostrum and in mature milk compared to those without GDM.^13^ Hence, stage of lactation, maternal BMI, and GDM appear to influence breast milk protein levels.

Another maternal characteristic that is worth investigating with regard to a possible influence on breast milk ovalbumin concentrations is maternal fish oil supplementation use. A 2018 systematic review and meta‐analysis identified that maternal fish oil supplementation may reduce the risk of infant egg sensitisation (6 trials, risk ratio 0.69, 95% CI 0.53–0.90).^14^ Interestingly, in a recent pig study, maternal fish oil supplementation during late gestation and early lactation was found to increase the protein content of colostrum and mature milk.^15^ Hence, we aimed to identify whether breast milk ovalbumin concentrations are associated with the maternal characteristics of BMI, GDM and fish oil supplementation use.

## 2 METHODS

### 2.1 Study design

Using data previously collected in a randomized controlled trial investigating the effects of maternal egg consumption during early lactation,^16^ this secondary analysis aimed to determine whether breast milk ovalbumin concentrations are associated with maternal characteristics. Written informed consent was obtained prior to study participation. The trial was approved by local ethical institutional review boards (Human Research Ethics Committees) at Princess Margaret Hospital (2060EP), St John of God Hospitals (#619): Murdoch, Mt. Lawley, and Subiaco, and South Metropolitan Hospital (P/13/45): Kaleeya, in Western Australia. The trial was registered with the Australian New Zealand Clinical Trials Registry (ACTRN12613000643774).

### 2.2 Study participants

The participants were recruited in pregnancy, and all had a history of medically diagnosed allergic disease (asthma, eczema/atopic dermatitis, allergic rhinitis or IgE‐mediated food allergy). Participants delivered their baby ≥36 weeks gestation, were planning to breastfeed, and did not have an egg allergy. The maternal characteristics data of interest for this analysis due to a potential influence on breast milk protein content (pre‐pregnancy BMI, GDM and pregnancy fish oil supplementation use) were collected at 36–40 weeks gestation. In this secondary analysis, we have only included maternal participants from the trial who were randomized to the high‐egg diet group (recommended consumption of more than four eggs per week) and the low‐egg diet group (recommended consumption of one to three eggs per week), who consumed one egg 2–6 h prior to collecting a breast milk sample, and who had collected a breast milk sample at all three timepoints of 2, 4, and 6 weeks of lactation.

### 2.3 Breast milk ovalbumin concentration measurements

Full details of the breast milk ovalbumin concentration measurements have been described previously.^16^ An Enzyme‐Linked Immunosorbent Assay (ELISA) kit (Alpha Diagnostic International, San Antonio, TX, USA) with an in‐house prepared control (ovalbumin 2 ng/mL, Sigma Aldrich, Castle Hill, NSW, Australia) was conducted according to the kit instructions. The ELISA had a detection range of 0.1–4.0 ng/mL, and the breast milk samples were run neat in triplicate. Any samples where the ovalbumin concentration was below the ELISA limit of detection were assigned to a value half that of the lowest limit of detection (0.05 ng/mL), consistent with the trial primary analysis of breast milk ovalbumin concentration measurements.^16^

### 2.4 Statistical analysis

Of the continuous data variables, maternal age was determined to be normally distributed, whereas birth gestation, pre‐pregnancy BMI, and breast milk ovalbumin concentrations were not normally distributed using the Kolmogorov–Smirnov test for normality. The Friedman Test was used to compare ovalbumin concentrations between the three time points (2, 4, and 6 weeks). The Mann–Whitney *U* test was used to compare the breast milk ovalbumin concentrations with the categorical maternal characteristics of GDM and pregnancy fish oil supplementation use. Spearman rho test was used to determine correlation coefficients between pre‐pregnancy BMI and breast milk ovalbumin concentrations. Data analysis was performed in IBM SPSS Statistics (Version 30.00). Figures were created using GraphPad Prism (Version 10.5.0).

## 3 RESULTS

### 3.1 Maternal characteristics

All 66 participants included in this analysis lived in Perth, Western Australia, and all had a history of allergic disease; 54 (82%) had allergic rhinitis, 37 (56%) had asthma, 26 (39%) had atopic dermatitis/eczema, and 8 (12%) had IgE‐mediated food allergy. Table 1 reports the participant characteristics. Sixteen (24.2%) women had taken fish oil supplementation in pregnancy, and six (9.1%) were diagnosed with GDM during pregnancy.

**TABLE 1:** Participant characteristics.

Maternal characteristic	Breast milk samples collected (*n* = 66)^c^	Pregnancy fish oil supplementation (*n* = 16)^d^	Gestational diabetes mellitus (*n* = 6)^e^
Allergic rhinitis	54 (81.8%)	15 (93.8%)	6 (100.0%)
Asthma	37 (56.1%)	5 (31.3%)	2 (33.3%)
Atopic dermatitis/eczema	26 (39.4%)	3 (18.8%)	3 (50.0%)
IgE‐mediated food allergy	8 (12.1%)	2 (12.5%)	0 (0.0%)
Caucasian descent	60 (90.9%)	13 (81.3%)	3 (50.0%)
Pre‐pregnancy body mass index (kg/m^2^)^b^	23.9 (22.0–26.2) (*n* = 62)	22.9 (21.0–26.7) (*n* = 16)	25.7 (22.2–37.6) (*n* = 6)
Age (years)^a^	33.4 (3.4)	34.2 (3.5)	33.1 (3.1)
Multiparous	8 (12.1%)	3 (18.8%)	2 (33.3%)
Birth mode: Cesarean‐section	27 (40.9%)	8 (50.0%)	4 (66.7%)
Birth gestation (weeks)^b^	39.7 (38.5–40.4)	39.6 (38.4–39.7)	39.1 (37.9–39.8)
Allocated to the high‐egg diet (>4 eggs/week) for trial intervention period	30 (45.5%)	6 (37.5%)	2 (33.3%)
Egg intake during week prior to sample collection (eggs/week)^b^	3.7 (2.3–4.9)	3.6 (2.1–5.1)	3.6 (2.2–4.2)

^a^ Mean (standard deviation).

^b^ Median (inter‐quartile range).

^c^ Participants who consumed one egg 2–6 h prior to collecting a breast milk sample, and who had collected a breast milk sample at all three timepoints of 2, 4, and 6 weeks of lactation.

^d^ Participants who took fish oil supplementation during pregnancy.

^e^ Participants who had gestational diabetes mellitus.

### 3.2 Breast milk ovalbumin concentrations

The median breast milk ovalbumin concentrations are reported in Table 2 and were not different when compared between time points (*p* = .08). Ovalbumin concentrations (all after consumption of one egg) were not different between the women allocated to the trial low‐egg group (*n* = 36) at 2 weeks = 0.19 (IQR 0.05–0.77) ng/mL, at 4 weeks = 0.05 (IQR 0.05–0.48) ng/mL and at 6 weeks = 0.05 (IQR 0.05–0.44) ng/mL, compared to the women allocated to the trial high‐egg group (*n* = 30) at 2 weeks = 0.16 (IQR 0.05–0.80) ng/mL (*p* = .94), at 4 weeks = 0.12 (IQR 0.05–0.74) ng/mL (*p* = .36), and at 6 weeks = 0.19 (IQR 0.05–1.13) ng/mL (*p* = .18).

**TABLE 2:** Breast milk ovalbumin concentrations (*n* = 66) at 2, 4, and 6 weeks of lactation.

	2 weeks	4 weeks	6 weeks	*p*‐value
Ovalbumin				
Median (Interquartile range)	0.18 ng/mL (0.05–0.78)	0.08 ng/mL (0.05–0.57)	0.08 ng/mL (0.05–0.54)	.08

**FIGURE 1:**
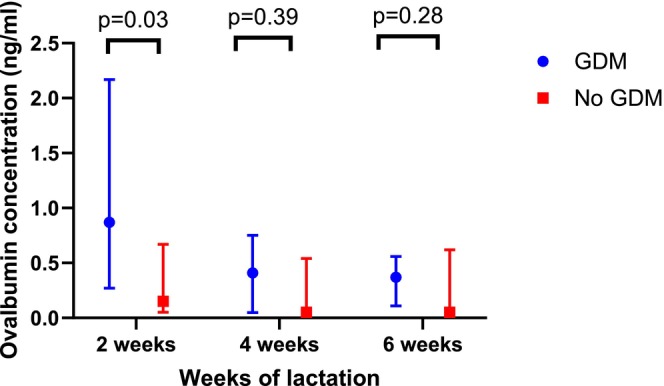
Breast milk ovalbumin concentrations (median and interquartile ranges) at 2, 4, and 6 weeks of lactation comparing the 6 women with gestational diabetes mellitus (GDM) to the 60 women without GDM.

**FIGURE 2:**
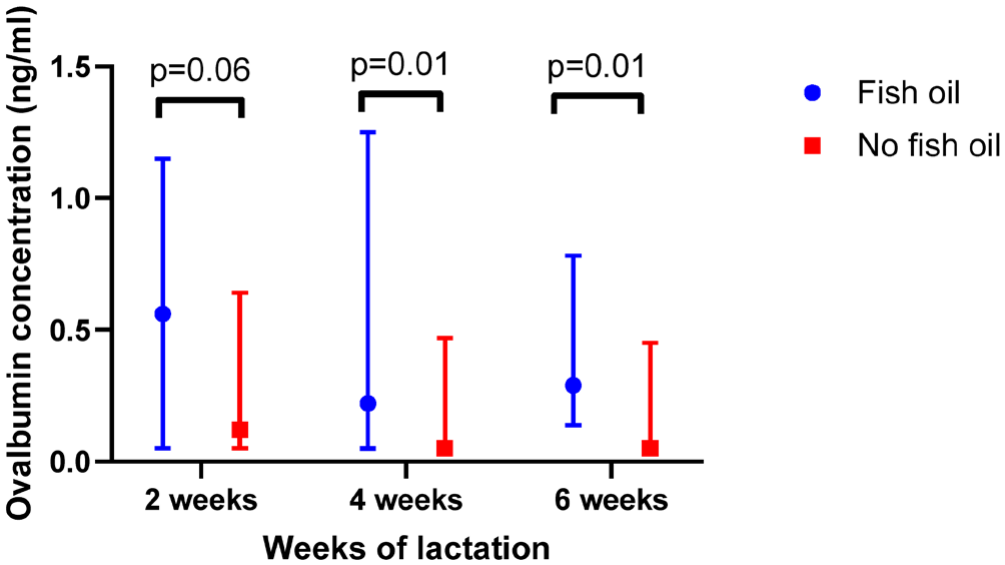
Breast milk ovalbumin concentrations (median and interquartile ranges) at 2, 4, and 6 weeks of lactation comparing the 16 women who took fish oil supplementation compared to the 50 women who did not take fish oil supplementation during pregnancy.

Table 3 reports the median and IQR breast milk ovalbumin concentrations at 2, 4 and 6 weeks of lactation comparing women with and without GDM, and women who took fish oil supplementation during pregnancy compared to those who did not. Figure 1 illustrates the higher ovalbumin concentrations measured at 2 weeks of lactation (*p* = .03) in participants with GDM (*n* = 6) compared to participants without GDM (*n* = 60). Figure 2 illustrates that higher breast milk ovalbumin concentrations were associated with fish oil supplement use in pregnancy (*n* = 16) at both 4 weeks (*p* = .01) and 6 weeks (*p* = .01).

**TABLE 3:** Breast milk ovalbumin concentrations median and interquartile ranges (IQR) at 2, 4, and 6 weeks of lactation comparing women with and without gestational diabetes mellitus, and women who took fish oil supplementation during pregnancy compared to those who did not.

Maternal characteristic	2 weeks lactation	4 weeks lactation	6 weeks lactation
Gestational diabetes mellitus (*n* = 6)	0.87 ng/mL (IQR 0.27–2.17)	0.41 ng/mL (IQR 0.05–0.75)	0.37 ng/mL (IQR 0.11–0.56)
No gestational diabetes mellitus (*n* = 60)	0.15 ng/mL (IQR 0.05–0.67)	0.05 ng/mL (IQR 0.05–0.54)	0.05 ng/mL (IQR 0.05–0.62)
Pregnancy fish oil supplementation (*n* = 16)	0.54 ng/mL (IQR 0.08–1.12)	0.39 ng/mL (IQR 0.07–1.20)	0.29 ng/mL (IQR 0.15–0.86)
No pregnancy fish oil supplementation (*n* = 50)	0.09 ng/mL (IQR 0.05–0.67)	0.05 ng/mL (IQR 0.05–0.44)	0.05 ng/mL (IQR 0.05–0.40)

Median pre‐pregnancy BMI (*n* = 62) was 23.9 (IQR 22.0–26.2) kg/m^2^, with no available pre‐pregnancy BMI data from four participants. Pre‐pregnancy BMI was not correlated with breast milk ovalbumin concentrations at 2 weeks (*n* = 62, *r* = −.023, *p* = .86), 4 weeks (*n* = 62, *r* = −.124, *p* = .34) or 6 weeks (*n* = 62, *r* = .010, *p* = .94).

## 4 DISCUSSION

The mechanism(s) enabling food protein passage into breast milk remain unknown. Our novel findings of higher breast milk ovalbumin concentrations in women who had GDM and those women who took a fish oil supplement during pregnancy begin to advance our knowledge of previously observed inter‐individual variations in food allergen concentrations in breast milk.

Fish oil supplements usually contain the omega‐3 fatty acid docosahexaenoic acid (DHA). Animal models have identified that DHA has been shown to aid mammary gland development,^17^ and supplementation during late gestation and early lactation was found to increase protein concentrations in pig milk.^15^ Fish oil supplementation during pregnancy and breastfeeding has also been associated with changes in breast milk immunoglobulin levels.^18^, ^19^ However, total protein concentrations in breast milk have not been shown to change in response to fish oil supplementation during pregnancy,^20^ and food allergen concentrations were not previously investigated. In human clinical trials, such as the randomized controlled trial (*n* = 706) by Palmer et al.,^21^ fish oil supplementation during pregnancy (including 800 mg DHA per day) was found to reduce the risk of infant egg sensitisation at 1‐year of age (adjusted relative risk 0.62, 95% confidence interval (CI) 0.41–0.93, *p* = .02). A systematic review and meta‐analysis of maternal fish oil supplementation trials determined that there was an overall reduced risk of infant egg sensitisation (6 trials, risk ratio 0.69, 95% CI 0.53–0.90),^14^ and interestingly also identified that supplementation during pregnancy appears more effective (4 trials, risk ratio 0.55, 95% CI 0.40–0.76) than during lactation (2 trials, risk ratio 0.92, 95% CI 0.65–1.28). In addition, this systematic review and meta‐analysis^14^ found that the risk of allergic sensitisation to peanut was also reduced with supplementation during pregnancy again appearing to be more effective (2 trials, risk ratio 0.62, 95% CI 0.40–0.96). Animal models have previously determined that increased oral exposure to food allergens through maternal milk reduced pup sensitisation to food allergens.^22^ Hence, we speculate that maternal fish oil supplementation may increase infant food allergen ingestion during lactation via higher breast milk food allergen concentrations, which may then lead to reduced infant food allergen sensitisation. Current human, large‐scale, randomized controlled trials are currently underway investigating whether the risk of developing food allergies in children can be reduced by maternal diets rich in food allergen consumption during lactation.^7^, ^8^ Our novel finding of higher ovalbumin concentrations in the breast milk of women who had taken fish oil supplementation during pregnancy may be an important maternal characteristic to consider in the future translation of these current trials.

Another maternal characteristic that is worthy of future investigation is our finding of higher breast milk ovalbumin concentrations in women who had GDM, although we do caution the overinterpretation of these findings given our study only included six women with GDM. Women with GDM have been found to have increased intestinal permeability.^23^ Hence, it is plausible that maternal diet food allergens may more readily pass through the gut barrier and into breast milk. As GDM resolves post‐birth, we speculate that the increased intestinal permeability may also resolve leading to the diminished differences in breast milk ovalbumin concentrations that we observed between women with and without GDM at 4 and 6 weeks compared to at 2 weeks of lactation. The critical period when higher breast milk food protein concentrations may have more influence on infant food allergy outcomes remains unknown. An interesting study by Chen and colleagues,^24^ identified that children of mothers with GDM were more likely to develop a food allergy, but that dietary polyunsaturated fatty acids, such as omega‐3 found in fish oil, might modify this association. Further investigations on the potential influences on breast milk food protein concentrations and infant food allergy outcomes of both GDM and maternal dietary intakes (including type and dose of supplementation use) of polyunsaturated fatty acids appear warranted.

Women who have a higher pre‐pregnancy BMI have an increased risk of developing GDM.^25^ Previous studies have identified higher protein concentrations in women with higher BMI,^10^, ^11^, ^12^ however, we found no associations between a higher pre‐pregnancy BMI and breast milk ovalbumin concentrations. One explanation for this could have been that the majority of the women participating in our study were in the healthy weight range with median pre‐pregnancy BMI = 23.9 kg/m^2^ (IQR 22.0–26.2). In comparison, the study by Liang et al.^12^ found higher breast milk protein concentrations in women who had a BMI ≥28 kg/m^2^, especially when compared to women who were underweight with a BMI <18.5 kg/m^2^ (*p* = .006). Hence, future studies investigating breast milk food allergen concentrations should include women with a wider BMI range.

A strength of this secondary analysis is the inclusion of participants who provided a breast milk sample at each of the three time points (2, 4, and 6 weeks of lactation) and on each occasion within 2–6 h of ingestion of one egg. This timing of breast milk sample collection was designed to measure ovalbumin concentrations when they are known to peak, as determined in a previous ovalbumin detection in breast milk study.^4^ However, the lack of a baseline (prior to egg consumption) breast milk sample and frequent repeated sampling after egg consumption could be considered a limitation of this study.

Another limitation of our study was that all participants had a history of allergic disease, hence we were unable to investigate any associations between breast milk ovalbumin concentrations and maternal atopy. In our recent systematic review,^2^ we identified four previous studies detecting food allergens (cow's milk and egg) in breast milk that were unable to find an association between maternal allergic disease and food allergen concentrations in breast milk. However, a newly published study investigating breast milk concentrations of the peanut protein, Ara h 2, found participants with a history of atopy were more likely to have a higher concentration of Ara h 2 in their breast milk, with median = 246 pg/mL (range 2.0–1634 pg/mL, *n* = 31) compared to non‐atopic mothers with median = 0 pg/mL (range 0–135, *n* = 8, *p* = .017).^25^ This newly published study^26^ also interestingly found that women who had detectable ovalbumin in their breast milk not always had detectable peanut protein (Ara h 2) and vice versa, despite eating both food allergens within the same meal prior to breast milk sample collection. Burris et al.^26^ suggest that this finding may indicate that there may be an allergen‐specific mechanism such as a specific food allergen antibody‐facilitated transfer. Therefore, future studies investigating food allergen concentrations in breast milk should aim to recruit both atopic and non‐atopic participants and investigate intra‐woman measurements of multiple food allergen concentrations in breast milk samples.

In conclusion, the mechanisms enabling food allergen protein transfer from the maternal diet into breast milk remain unknown. Our new findings indicate that the effects of fish oil supplementation and GDM on food protein concentrations in breast milk require further investigation. Gaining a deeper understanding of the maternal characteristics that influence breast milk food allergen concentrations could lead to future personalized approaches to maternal diet during lactation intervention strategies for infant food allergy prevention.

## AUTHOR CONTRIBUTIONS

**Debra J. Palmer:** Conceptualization; funding acquisition; writing – review and editing; validation; methodology; formal analysis; project administration; supervision; resources. **Jessica R. Metcalfe:** Investigation; methodology; data curation; writing – review and editing. **Sophie A. Hughes:** Investigation; methodology; writing – original draft; formal analysis; data curation. **Donna T. Geddes:** Writing – review and editing; supervision. **Sharon L. Perrella:** Writing – review and editing; supervision.

## FUNDING INFORMATION

The study was supported by a National Health and Medical Research Council (NHMRC) Project Grant (ID 1046036). SH was supported by an Australian Government Research Training Program Domestic Fees Offset Scholarship, an Australian Government Research Training Stipend and a National Health and Medical Research Council Centre of Research Excellence (ID 2015724) Centre for Food Allergy Research (CFAR) Postgraduate Top‐up Scholarship. DG and SP are supported by an unrestricted research grant from Medela AG, administered by The University of Western Australia. DP was supported by a Stan Perron Charitable Foundation Fellowship.

## CONFLICT OF INTEREST STATEMENT

All authors declare no conflict of interest.

## Data Availability

The data that support the findings of this study are available from the corresponding author upon reasonable request.
